# Quantitative ethnobotany and evidence-based analysis of antidiabetic medicinal plants used in Biswanath district, Assam, India

**DOI:** 10.1038/s41598-026-70735-8

**Published:** 2026-09-08

**Authors:** Bichitra Gohain, Rishab Sharma, Helen Soibam, Victor Singh Ayam

**Affiliations:** 1 Department of Botany, Rajiv Gandhi University, Doimukh, 791112 Arunachal Pradesh India; 2 Central Agricultural University, Imphal, College of Agriculture, AICRP-MAP&B, Pasighat, Arunachal Pradesh India

**Keywords:** Ethnobotany, Antidiabetic plants, Cultural salience, Evidence mapping, Northeast India, Health care, Plant sciences

## Abstract

Type 2 diabetes is a growing public health concern in India, while plant-based remedies remain important in Assam. This study documented plants used for diabetes-related complaints in Biswanath district, quantified diabetes-specific salience and consensus, and integrated ethnobotanical indices with pharmacological evidence. From December 2022 to March 2024, semi-structured interviews with 440 informants were complemented by voucher-based taxonomic authentication. Relative Frequency of Citation (RFC) and Fidelity Level (FL) were diabetes-specific, whereas Use Value (UV) measured ethnomedicinal versatility across all ailments. Published species-level antidiabetic evidence was graded as no eligible evidence, in vitro only, or in vivo and/or human. We documented 63 species in 57 genera and 39 families. Among 83 plant-part use records, fruit (22.9%) and leaf (21.7%) were most frequent. *Andrographis paniculata* (RFC = 0.223), *Kalanchoe pinnata* (0.200), and *Nyctanthes arbor-tristis* (0.180) were most frequently cited for diabetes; *Allium sativum* had the highest overall UV across all ailments (2.359), and *Syzygium cumini* the highest diabetes-specific FL (92.59%). Evidence–salience mapping placed 16 species (25.4%) in the Overlap zone; none met Gap-zone criteria under the species-level framework. *Hydrocotyle sibthorpioides* and *Alocasia odora* had only in vitro evidence. The framework supports prioritisation but does not establish the efficacy or safety of locally used plant parts or preparations.

## Introduction

Ethnomedicinal plant knowledge remains a primary healthcare resource across rural Assam and the wider Indo–Burma region, supported by high floristic diversity and strong community practice. Type 2 diabetes mellitus (T2DM) is a rapidly escalating metabolic disorder and a major contributor to global morbidity and mortality, with the greatest burden borne by low- and middle-income countries^1,2^. The burden of type 2 diabetes in India has increased markedly over time and is projected to rise further in the coming decades, reflecting a sustained upward trend in prevalence and public health impact^3–5^. Although multiple oral hypoglycaemic agents and insulin formulations are available, long-term diabetes management remains constrained by cost, limited accessibility, adverse effects, and interindividual variability in therapeutic response^6–8^. These limitations reinforce reliance on medicinal plants in South and Southeast Asia, where plant-based therapies are deeply embedded in traditional medical systems^9,10^. Here, diabetes-related remedies are examined as a focal use-category within broader ethnomedicinal knowledge, rather than as a disease-specific inventory.

India is a global centre of traditional medical knowledge, with Ayurveda, Siddha, and Unani systems documenting extensive plant-based therapies for metabolic disorders, including conditions broadly corresponding to diabetes (Prameha/Madhumeha)^11,12^. Northeast India lies within the Indo–Burma biodiversity hotspot and combines exceptional floristic richness with high cultural and ethnic diversity, resulting in highly localised ethnomedicinal traditions^13,14^. In Assam’s Brahmaputra valley, medicinal plants remain integral to household care for chronic metabolic conditions; however, this locally held knowledge is still under-documented and only partially integrated with contemporary pharmacological literature^15,16^.

Although a growing body of ethnobotanical literature from Northeast India and other regions has documented medicinal plants used in diabetes management^17–22^, most studies remain largely descriptive species inventories, with limited quantitative assessment of cultural importance and minimal integration with graded biomedical evidence^15,16,22^. Similar patterns have been reported from other geographical regions, including Mauritius, Uganda, and Pakistan, where traditional plant-based therapies continue to play a significant role in diabetes management^23–25^.

Evidence-mapping approaches help address this gap by linking traditional use patterns with quantitative consensus indices and evidence-graded synthesis of pharmacological support, thereby enabling prioritisation of taxa for downstream phytochemical and mechanistic investigation^26–28^. Many plant secondary metabolites—including flavonoids, terpenoids, phenolic acids, alkaloids, and saponins—have been associated with glycaemic regulation through multiple mechanisms, including enzyme inhibition and modulation of oxidative and insulin-related pathways^29,30^. Accordingly, assessing concordance between cultural salience and evidence strength is critical for transparent prioritisation of candidate taxa for follow-up investigation.

Biswanath district in Assam is inhabited by ethnically diverse communities—including Assamese, Nepali, Mising, Karbi, Boro, and Adivasi groups—who use a wide range of wild and cultivated plants in household healthcare, including remedies for diabetes-related metabolic conditions. However, studies from this region have not yet combined quantitative salience metrics with evidence-graded mapping of culturally important taxa, and concordance between community consensus and evidence strength remains insufficiently characterised. We therefore hypothesise that species with higher diabetes-specific cultural salience and consensus, as reflected by RFC and FL, are more likely to align with existing pharmacological evidence for antidiabetic activity, while also highlighting culturally important taxa that remain insufficiently explored.

To address this gap, we assess ethnomedicinal plant knowledge in Biswanath district, Assam, with diabetes-related uses analysed in depth. Specifically, we (i) document plants used traditionally for diabetes management; (ii) quantify diabetes-specific cultural salience and consensus using Relative Frequency of Citation (RFC) and Fidelity Level (FL), and assess overall ethnomedicinal versatility across recorded ailments using Use Value (UV); and (iii) map the strength of published pharmacological evidence (in vitro, in vivo, and human studies). By integrating quantitative ethnobotany with evidence grading, we generate a transparent priority set of taxa to guide follow-up testing, phytochemical characterisation, and mechanistic investigation, while supporting community-aligned research and conservation planning in Northeast India.

We combined field-based ethnomedicinal documentation with quantitative indices (RFC, UV, FL) and an evidence-graded literature synthesis to generate a reproducible and transparent prioritisation framework. The following section describes the study area, sampling and voucher procedures, quantitative analyses, and the criteria used to grade published pharmacological evidence.

## Materials and methods

This study in Biswanath district, Assam, India, comprised (i) ethnobotanical field surveys with voucher-based plant authentication and (ii) quantitative analysis of cultural salience and evidence-graded mapping of diabetes-related uses.

### Study area

**Fig. 1:**
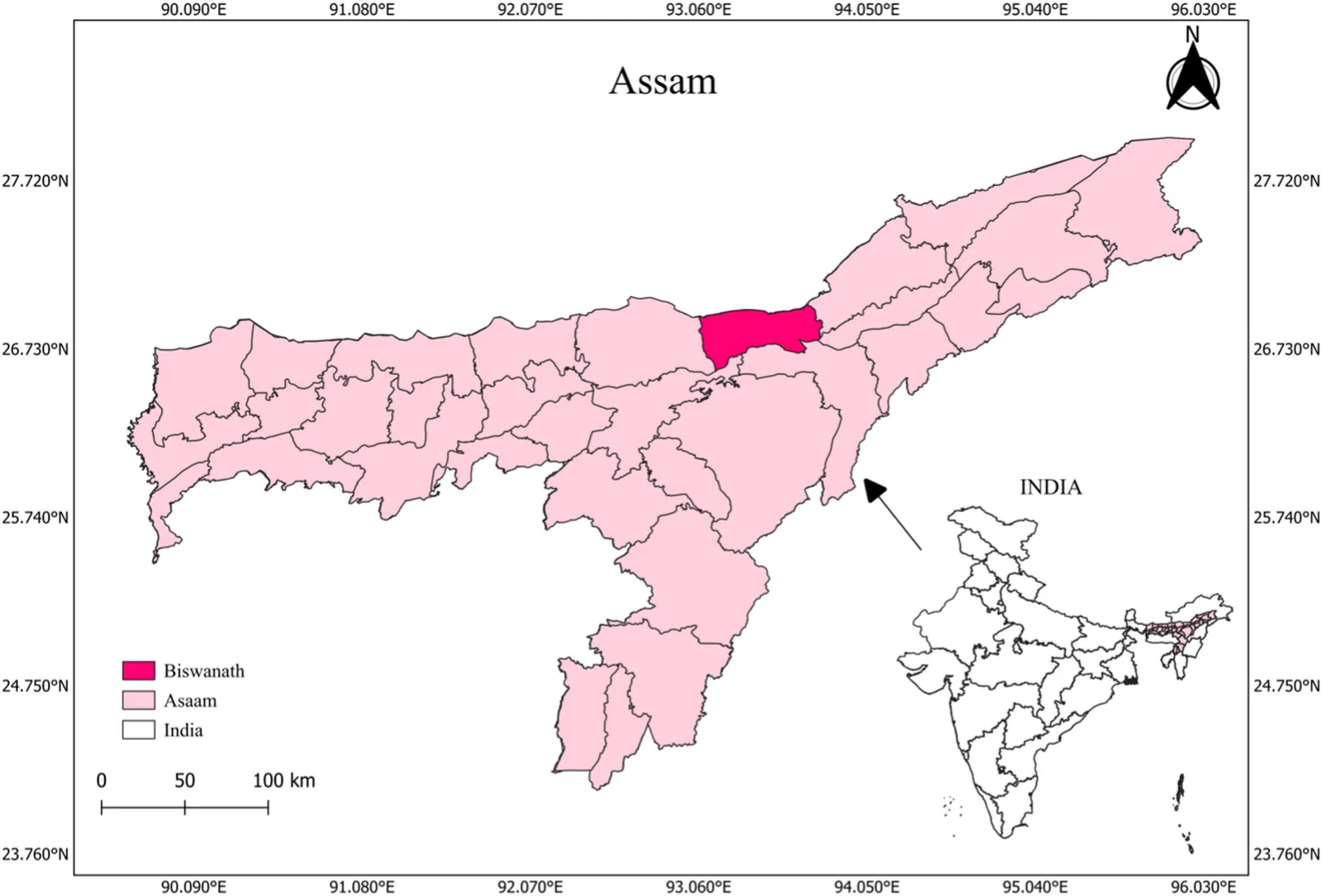
Location map of the study area in Biswanath district, Assam, India. The map was created by the authors using QGIS version 3.32 (QGIS Geographic Information System; https://www.qgis.org/download/) with India ADM1 and ADM2 administrative boundary data obtained from geoBoundaries (https://www.geoboundaries.org/), available under the Creative Commons Attribution 4.0 International (CC BY 4.0) licence. Boundary data source: Runfola et al.^31^.

Biswanath district is located on the northern bank of the Brahmaputra River within the Indo–Burma biodiversity hotspot. It lies between 92°16′–93°43′E and 26°30′–27°01′N (Fig. 1), covers approximately 1,415 km^2^, and spans 48–849 m in elevation. The district has a subtropical, monsoon-influenced climate and comprises a mosaic of forest fragments, agricultural lands, riverine sandbars (chars), and village home gardens that collectively support diverse medicinal plant resources.

### Ethical considerations and informed consent

Ethical approval for the interview-based ethnobotanical survey was obtained from the Institutional Ethical Committee, Rajiv Gandhi University, Arunachal Pradesh, India (F. No. RGU/EO-55/IEC/2022/). Institutional permission to conduct the study was granted by Rajiv Gandhi University (Memo No. REGN-4075/2022/105; 16 December 2022). The study involved non-interventional ethnobotanical interviews with adult participants. Participation was voluntary, and prior informed consent (written or verbal, as appropriate) was obtained. Data were anonymised, and no personally identifiable information was disclosed. The study was conducted in accordance with the Code of Ethics of the International Society of Ethnobiology and the principles of the Convention on Biological Diversity (1992) and the Nagoya Protocol (2010).

### Informant selection

Fieldwork was conducted from December 2022 to March 2024. Informants were recruited using purposive sampling, complemented by snowball referrals to reach participants familiar with local medicinal plant practices. All participants were adults. In total, 440 informants (276 men and 164 women; 25–84 years) representing Assamese, Nepali, Mising, Karbi, Boro, Adivasi, and other local communities were interviewed. Participants included traditional health practitioners, elderly knowledge holders, household caregivers, and other community members. Socio-demographic characteristics were recorded for each informant (Table 1). Formal data saturation was not assessed as a stopping criterion; recruitment continued throughout the planned field-survey period.

### Ethnomedicinal data collection

**Fig. 2:**
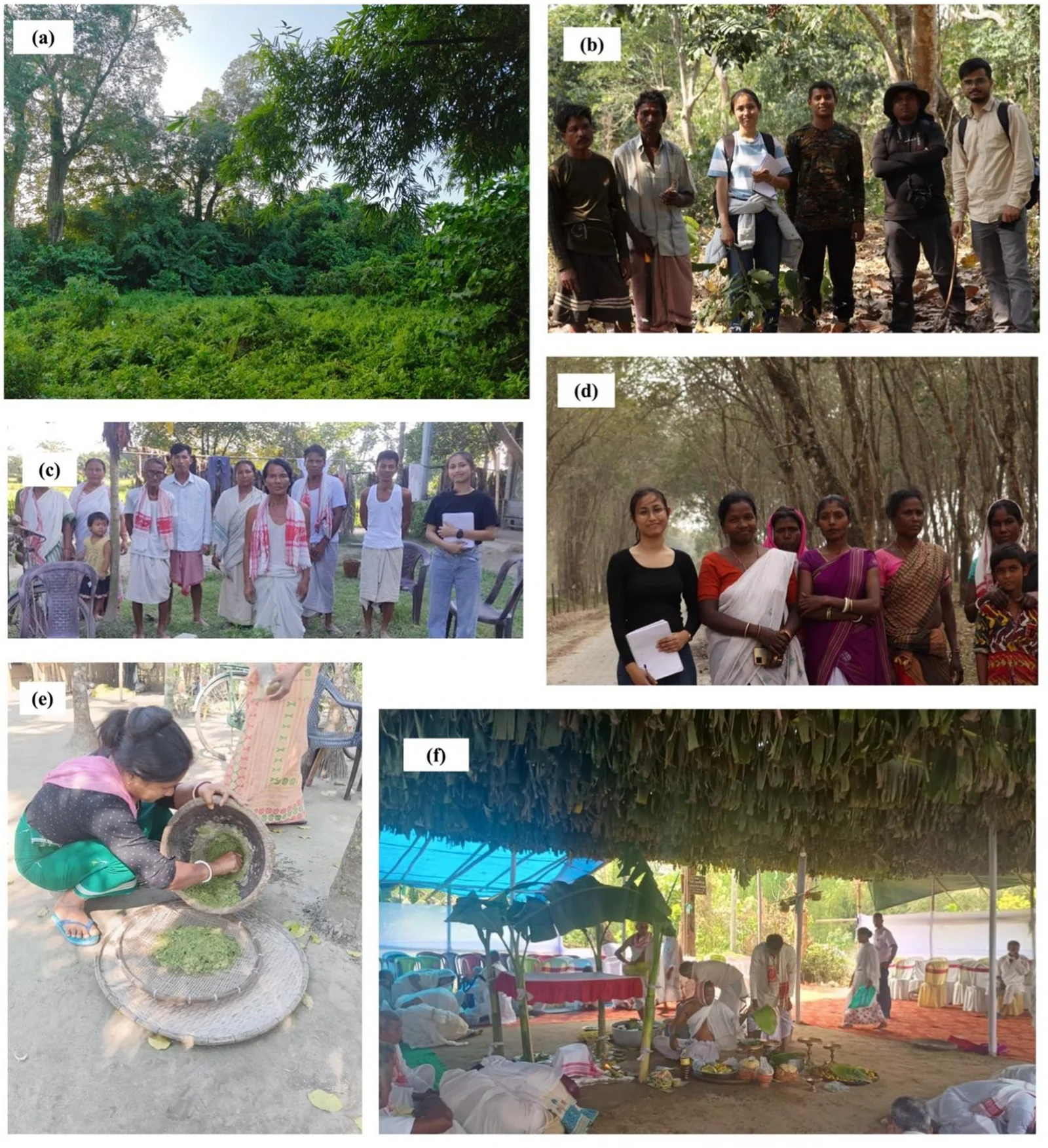
(**a**–**f**) Representative landscapes and ethnobotanical documentation of Biswanath district, Assam. (**a**) Landscape of the study area; (**b**) field survey of medicinal plants; (**c**) interaction with male informants during ethnobotanical interviews; (**d**) interaction with female informants documenting knowledge transmission; (**e**) Traditional preparation of antidiabetic remedies by local practitioners; (**f**) socio-cultural rituals (marriage ceremony) involving symbolic and medicinal plants.

**Fig. 3:**
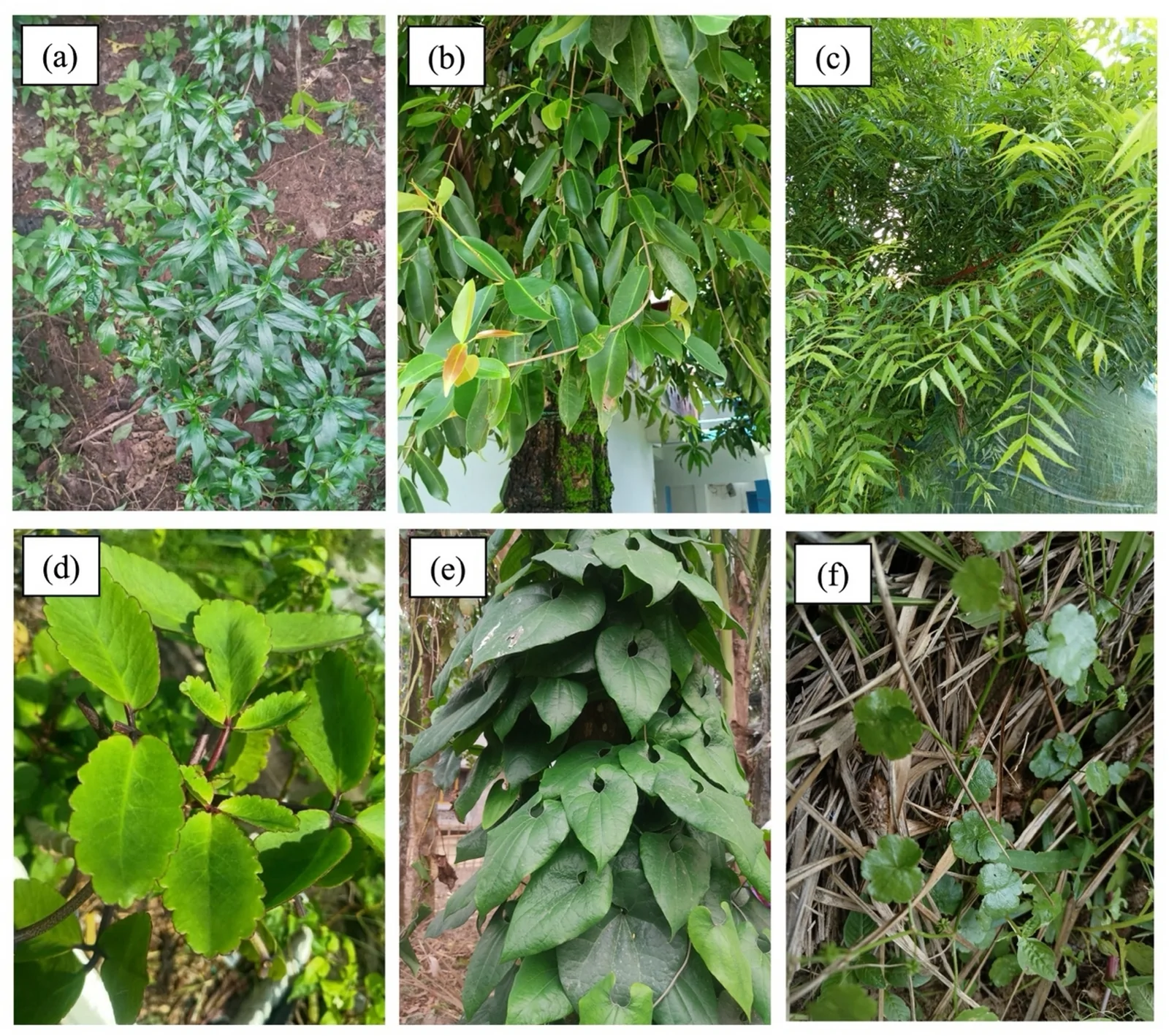
(**a**–**f**) Representative antidiabetic plant species documented from Biswanath district, Assam. (**a**) *Andrographis paniculata* (Kalmegh); (**b**) *Syzygium cumini* (Jamun); (**c**) *Azadirachta indica* (Neem); (**d**) *Kalanchoe pinnata* (Patharkuchi); (**e**) *Aristolochia assamica* (local ethnomedicinal climber); (**f**) *Hydrocotyle sibthorpioides.*

Ethnomedicinal information on plants used for diabetes-related complaints (often referred to locally as “high sugar”) was collected through semi-structured interviews, free-listing, and guided field walks. Interviews were conducted in Assamese and/or local vernaculars, with assistance from local translators when required. For each reported species, we recorded local name(s), plant part(s) used, preparation method, dosage form, route of administration, frequency and duration of use, and any reported adverse effects or precautions. During field walks, plants were observed in situ and linked to voucher specimens for subsequent authentication. Key details were verified through repeat discussions where feasible and cross-checking across informants and field observations^32,33^. Representative field settings and selected documented taxa are shown in Figs. 2 and 3.

### Plant collection, voucher preparation, and identification

Voucher specimens were collected during guided field walks based on informant reports. Permission for collection was obtained from private landholders and from village authorities for community lands; sampling followed local norms and minimal-impact methods. For herbs, whole plants were collected where feasible, whereas for shrubs and trees flowering and/or fruiting twigs were collected to facilitate accurate identification. Specimens were pressed, dried, and mounted following standard herbarium procedures^34^.

Formal identification of the plant material was undertaken by Dr. Victor Singh Ayam (Department of Botany, Rajiv Gandhi University, Arunachal Pradesh, India) using regional floras and standard taxonomic keys, including *The Flora of Assam* and *Flora of British India*^35,36^. Scientific names, accepted names and author citations were standardised according to Plants of the World Online (POWO), with World Flora Online (WFO) consulted for cross-verification. Voucher specimens were deposited in the publicly accessible herbarium of the Department of Botany, Rajiv Gandhi University (HAU; an institutionally accepted local acronym retained from the University’s former name, Arunachal University, and not used here as an Index Herbariorum code), with accession numbers HAU/AN-3780–HAU/AN-3842, as listed in Table 2. Identification was further confirmed by Prof. Abhaya Prasad Das (Retired Professor of Botany, University of North Bengal, Siliguri, West Bengal, India).

### Quantitative ethnobotanical analysis

Ethnomedicinal data comprised informant-level citations of species for diabetes and other reported ailments. RFC was calculated from diabetes-specific informant citations, FL from diabetes-specific and all-use informant counts, and UV from use-reports across all recorded ailments.

#### Relative frequency of citation (RFC)

Relative Frequency of Citation was calculated for each species following established quantitative ethnobotanical protocols^37^ as:

$$\:RFC=\frac{FC}{N}\hspace{1em}\left(0<RFC\le\:1\right)$$

where FC is the number of informants who cited a species for diabetes management and N is the total number of informants interviewed (*N* = 440). Higher RFC values indicate greater cultural salience.

#### Fidelity level (FL)

To assess consensus on the use of a species for diabetes relative to all reported uses of that species, Fidelity Level was computed^38^ as:

$$\:FL\left(\%\right)=\frac{{N}_{p}}{{N}_{u}}\times\:100$$

where N_p_ is the number of informants citing the species specifically for diabetes and *N*_*u*_ is the total number of informants citing the same species for any use category or ailment.

#### Use value (UV)

Overall ethnomedicinal versatility across all recorded ailments was estimated using Use Value (UV)^39^ as:

$$\:\mathrm{U}{V}_{s}=\frac{{\sum\:}_{i=1}^{N}{U}_{is}}{N}$$

where *U*_*is*_ is the number of use-reports across all recorded ailments mentioned by informant *i* for species *s*, and *N* is the total number of informants interviewed (*N* = 440).

Collectively, RFC reflects diabetes-specific cultural salience, FL indicates diabetes-specific informant consensus relative to all reported uses of a species, and UV captures overall ethnomedicinal versatility across all recorded ailments. UV should therefore not be interpreted as a measure of antidiabetic importance. The combined interpretation of these indices enables discrimination between widely cited multipurpose species and taxa showing high specificity for diabetes management, thereby strengthening prioritization for downstream pharmacological evaluation.

### Pharmacological evidence mapping and prioritisation

To assess the extent of published pharmacological support for the recorded diabetes-related uses, we conducted a structured literature search for each documented species in PubMed, Scopus, Web of Science, and Google Scholar. Searches were performed using the accepted botanical name and relevant synonyms, combined with keywords including *antidiabetic*, *antihyperglycaemic*, α-glucosidase, α-amylase, insulin, glucose uptake, and oxidative stress. The structured literature search covered publications available up to 31 March 2024; targeted supplementary searches were subsequently conducted to identify relevant recent studies, with the final update completed on 1 August 2026. Non-English articles with English abstracts were also screened for eligibility. Retrieved records were screened for relevance to glycaemic control and included only studies with interpretable endpoints related to glucose homeostasis. Eligible evidence was classified by study type as: (i) in vitro (enzyme inhibition and/or relevant cell-based assays), (ii) in vivo (antihyperglycaemic/antidiabetic animal models), or (iii) clinical evidence (human intervention and/or observational studies).

For evidence-tier assignment, evidence was assessed at the species level; therefore, a published antidiabetic study meeting the stated eligibility criteria for any plant part or preparation of a species was sufficient for classification, whether or not it matched the locally documented remedy. Each species was assigned an evidence tier based on the highest level of support identified: E = 0 (no eligible antidiabetic evidence located), E = 1 (in vitro evidence only), and E = 2 (in vivo and/or human evidence). For prioritisation, Relative Frequency of Citation (RFC) was integrated with evidence tiers to identify culturally salient taxa supported by stronger evidence (overlap/priority set) and to flag culturally salient taxa with limited evidence as candidates for follow-up validation. For detailed visualisation in Fig. 7(b–e), taxa were first restricted to evidence tier E = 2 and then ranked according to RFC. The ten highest-RFC taxa within E = 2 were selected; thus, evidence tier served as the primary eligibility criterion, while RFC served as the secondary cultural-salience criterion. The resulting prioritisation map is presented in the Results section.

## Results

### Informant characteristics

A total of 440 informants were interviewed (276 men, 62.73%; 164 women, 37.27%), aged 25–84 years. The most represented age group was 55–64 years (*n* = 144; 32.73%), followed by 65–74 years (*n* = 110; 25.00%) and 45–54 years (*n* = 104; 23.64%) (Table 1). With respect to education, 28.41% (*n* = 125) reported no formal schooling, 38.18% (*n* = 168) completed primary education, 20.00% (*n* = 88) completed secondary education, and 13.41% (*n* = 59) had higher education (Table 1). This demographic profile reflects the diversity of knowledge holders contributing to the documentation of antidiabetic medicinal plants in Biswanath district.

**Table 1:** Demographic characteristics of informants.

Attribute	Group	No. of informants (*n*)	Percentage (%)
Gender	Men	276	62.73
	Women	164	37.27
Age (years)	25–34	14	3.18
	35–44	58	13.18
	45–54	104	23.64
	55–64	144	32.73
	65–74	110	25.00
	75–84	10	2.27
Education	No formal schooling	125	28.41
	Primary	168	38.18
	Secondary	88	20.00
	Higher	59	13.41
Total		440	100.00

### Diversity of antidiabetic medicinal plants

**Fig. 4:**
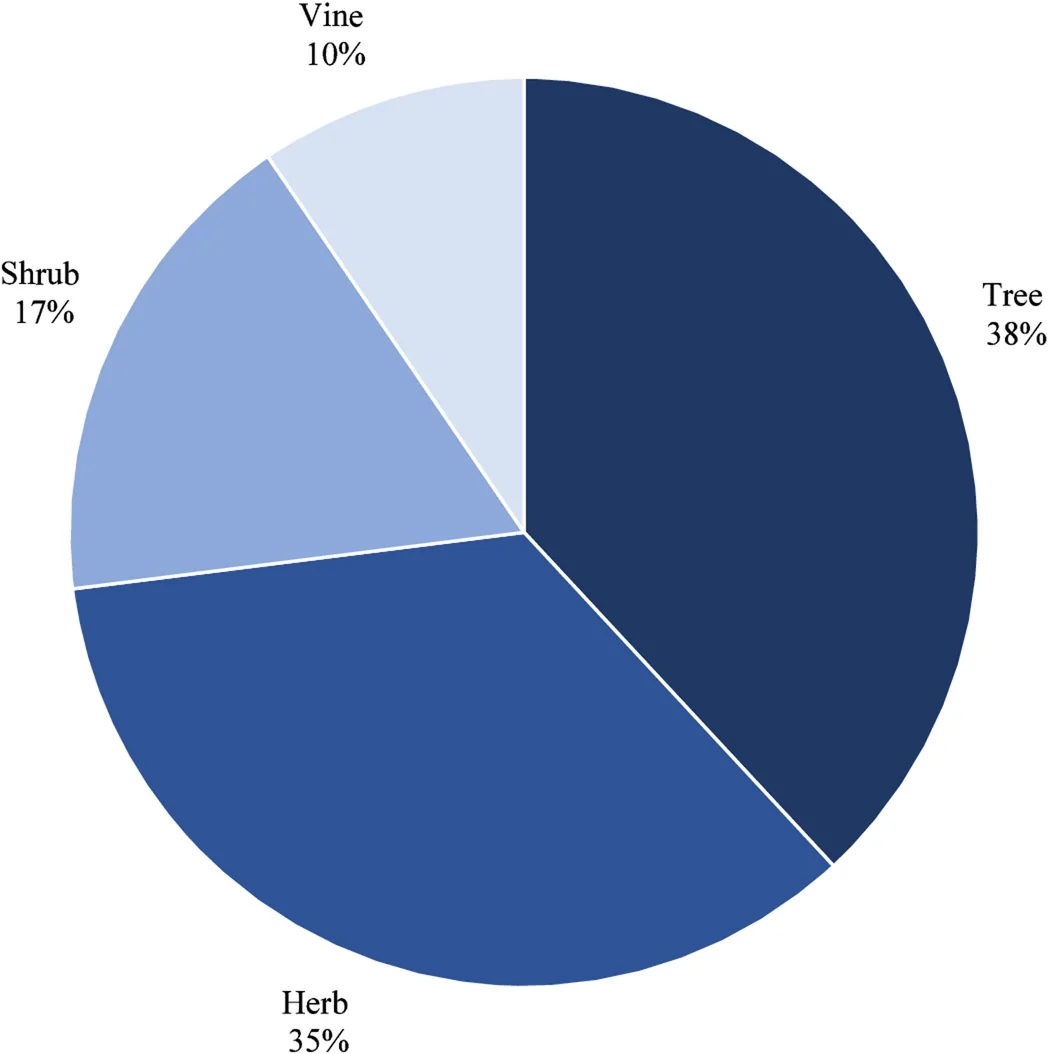
Growth-form composition of the 63 antidiabetic medicinal plant species documented in Biswanath district, Assam, India (tree, herb, shrub, vine).

**Fig. 5:**
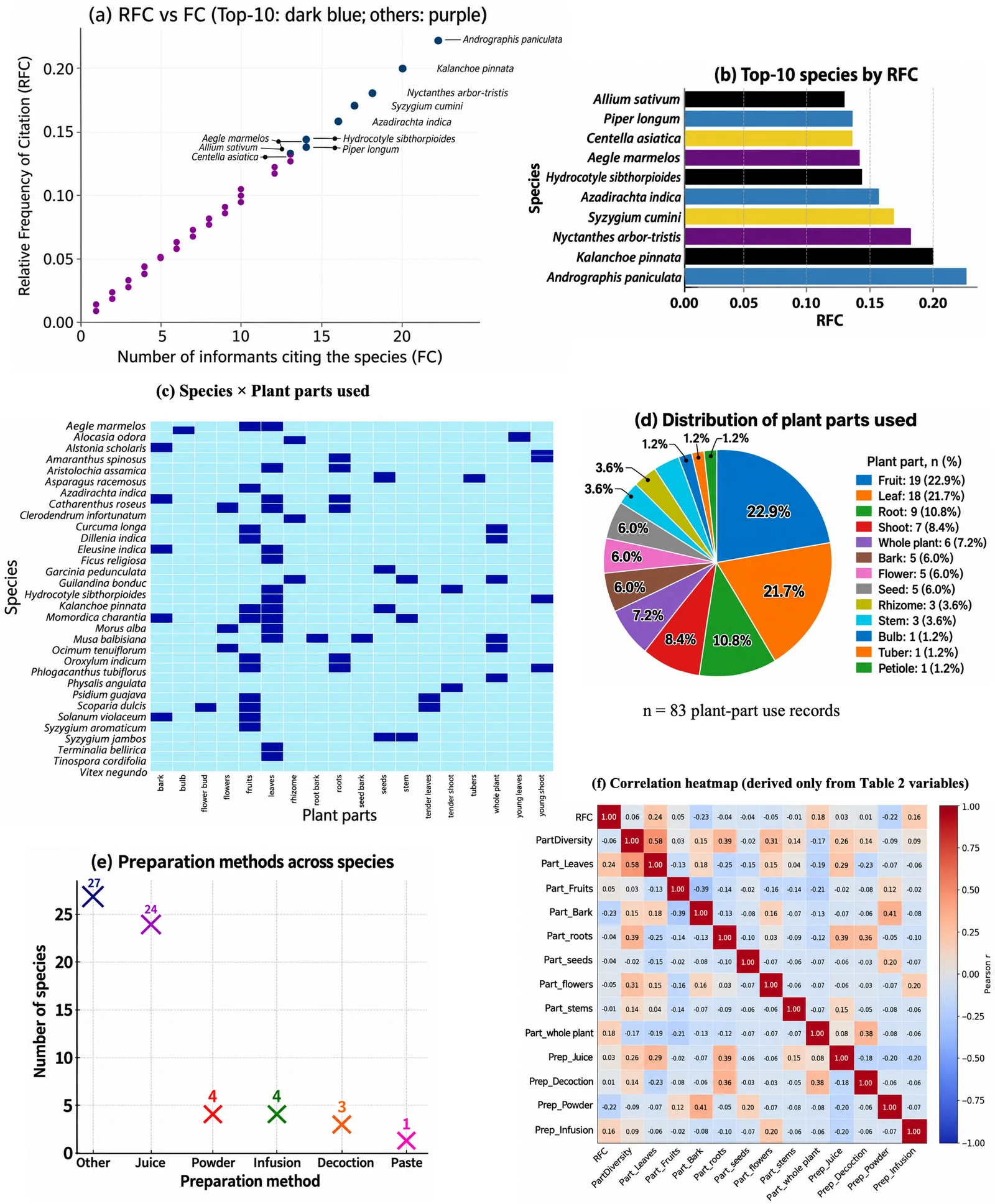
Quantitative summaries of the ethnobotanical data on antidiabetic plants documented in Biswanath district, Assam. (**a**) Scatter plot of Relative Frequency of Citation (RFC) against Frequency of Citation (FC), where FC denotes the number of informants citing each species. The 10 species with the highest RFC values—*Andrographis paniculata*, *Kalanchoe pinnata*, *Nyctanthes arbor-tristis*, *Syzygium cumini*, *Azadirachta indica*, *Hydrocotyle sibthorpioides*, *Aegle marmelos*, *Centella asiatica*, *Piper longum*, and *Allium sativum*—are highlighted in dark blue, while the remaining species are shown in purple. (**b**) Horizontal bar chart presenting the same 10 species in descending order of RFC. (**c**) Binary presence–absence heatmap showing the plant parts reported as used for a selected subset of species; the subset was displayed to reduce visual congestion, while the complete species–plant-part information is provided in Table 2. (d) Pie chart showing the distribution of 83 plant-part use records compiled from informant reports for the 63 documented species. Each distinct plant part reported for a species was counted separately. Related terms were standardised as follows: tender leaves as leaf, young and tender shoots as shoot, root bark as root, seed bark as seed, flower bud as flower, and young petiole as petiole. Percentages were calculated using 83 as the denominator and rounded to one decimal place. (e) Scaled-marker plot showing the number of species recorded under each preparation-method category, with the corresponding counts indicated above the markers. (f) Heatmap of pairwise Pearson correlation coefficients among the Table 2 variables shown, including RFC, plant-part diversity, plant-part presence–absence indicators, and preparation-method indicators.

Sixty-three antidiabetic plant species representing 57 genera and 39 families were documented (Table 2). The study area and field documentation are shown in Figs. 1, 2 and 3. Based on growth form, trees constituted the largest proportion of recorded taxa (38%), followed by herbs (35%), shrubs (17%) and vines (10%) (Fig. 4). Percentages in Figs. 4 and 5 are expressed as proportions of the recorded species pool (*n* = 63). Overall, the documented taxa indicate considerable taxonomic diversity in the antidiabetic medicinal flora reported by local informants.

### Plant parts used and modes of preparation

A total of 83 plant-part use records were compiled from informant reports for the 63 documented species. Fruit (19/83; 22.9%) and leaf (18/83; 21.7%) were the most frequently reported categories, followed by root (9/83; 10.8%), shoot (7/83; 8.4%), whole plant (6/83; 7.2%), bark, flower and seed (5/83 each; 6.0%), rhizome and stem (3/83 each; 3.6%), and bulb, tuber and petiole (1/83 each; 1.2%) (Fig. 5d). Where more than one plant part was reported for a species, each part was counted separately. Related terms were grouped into the broader categories described in the Fig. 5 caption. Figure 5(c) displays a subset of species to reduce visual congestion, while the complete species–plant-part information is provided in Table 2.

The preparation-method distribution comprised the grouped category “Other” (27 species), followed by juice (24 species), powder and infusion (4 species each), decoction (3 species), and paste (1 species) (Fig. 5e).

### Culturally salient taxa based on relative frequency of citation

Relative Frequency of Citation (RFC; Table 2) identified *Andrographis paniculata* as the most frequently cited antidiabetic species (RFC = 0.223), followed by *Kalanchoe pinnata* (0.200), *Nyctanthes arbor-tristis* (0.180), *Syzygium cumini* (0.170), and *Azadirachta indica* (0.157) (Fig. 5a,b). Other high-ranking taxa included *Hydrocotyle sibthorpioides*, *Aegle marmelos*, *Centella asiatica*, and *Piper longum*, while *Allium sativum* and *Phyllanthus emblica* had identical RFC values (0.132), resulting in a tie at the cut-off for the tenth position (Fig. 5a,b). Species with lower RFC values were cited by fewer informants in the present dataset. Correlation analysis among plant-part use, preparation modes, and citation frequency (Fig. 5f) did not indicate a strong association between RFC and part-use diversity. Instead, the heatmap highlights patterns of plant-part utilisation and preparation practices in local ethnomedicinal knowledge. Overall UV values across all recorded ailments and diabetes-specific FL values are reported in Table 2.

### Priority mapping using cultural salience and evidence tiers

**Fig. 6:**
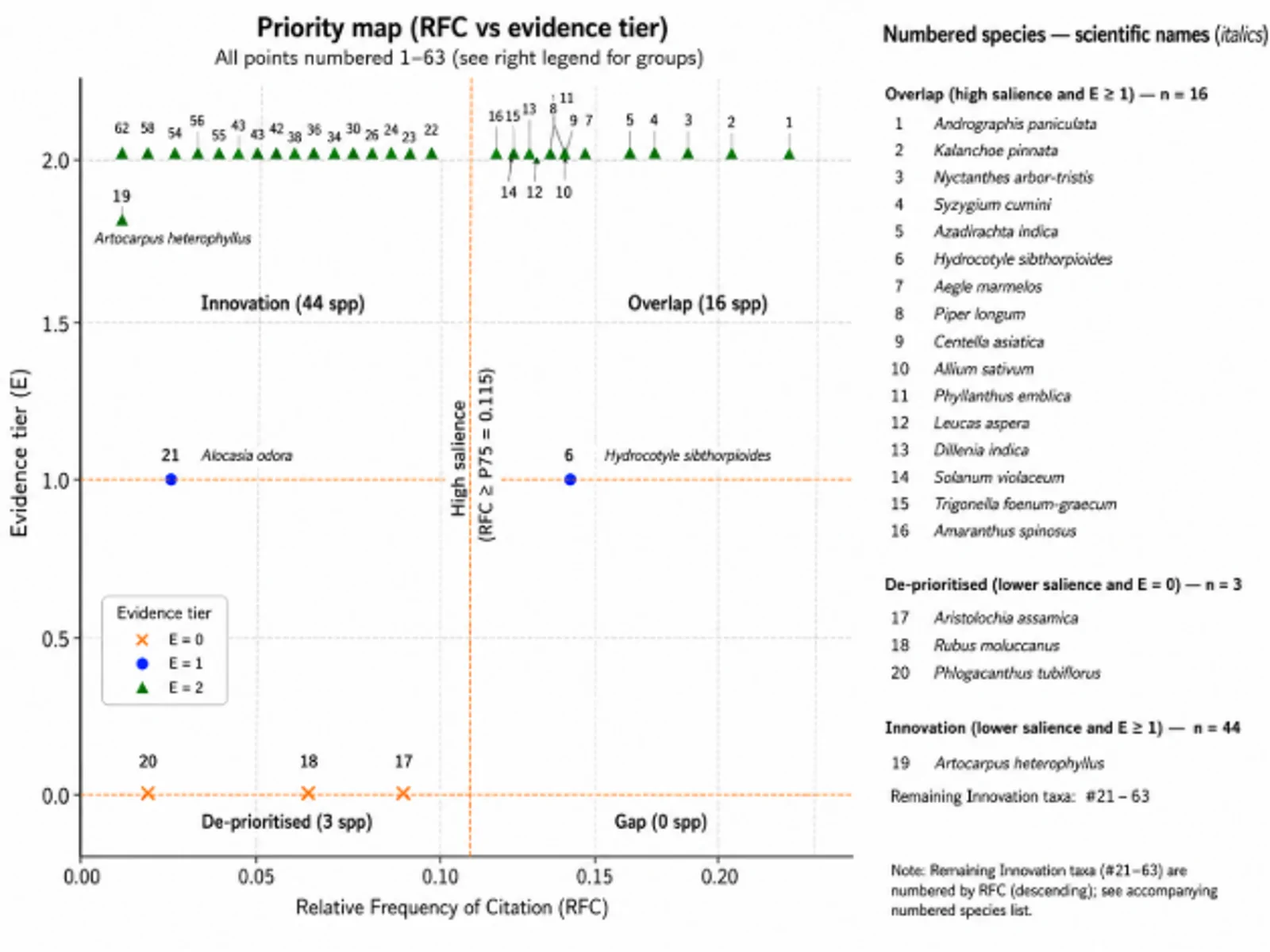
Priority map of Relative Frequency of Citation (RFC) against evidence tier for the 63 documented antidiabetic species. The x-axis shows RFC, and the y-axis shows evidence tier (E: 0 = no eligible antidiabetic evidence identified, 1 = in vitro evidence only, and 2 = in vivo and/or human evidence). High salience was defined as RFC ≥ P75, with the vertical dashed line indicating the P75 threshold (RFC = 0.115). Species were assigned to four zones: Overlap (high salience, E ≥ 1), Gap (high salience, E = 0), Innovation (lower salience, E ≥ 1), and De-prioritised (lower salience, E = 0). Points are numbered 1–63, with selected taxa identified in the right-hand panel. Zone totals were: Overlap = 16, Innovation = 44, De-prioritised = 3, and Gap = 0. The only two E = 1 taxa were *Hydrocotyle sibthorpioides* (#6; high salience) and *Alocasia odora* (#21; Innovation). *Artocarpus heterophyllus* (#19) was assigned to E = 2 and placed in the Innovation zone. The De-prioritised taxa were *Aristolochia assamica* (#17), *Rubus moluccanus* (#18), and *Phlogacanthus tubiflorus* (#20).

The RFC × evidence-tier framework (Fig. 6) classified all 63 documented species using RFC ≥ P75 (upper quartile) as the high-salience threshold and evidence tiers defined as E = 0 (no eligible antidiabetic evidence located), E = 1 (in vitro evidence only), and E = 2 (in vivo and/or human evidence). Species were mapped into four zones: Overlap (high salience; E ≥ 1), Gap (high salience; E = 0), Innovation (lower salience; E ≥ 1), and De-prioritised (lower salience; E = 0).

Overall, 16 species (25.4%) fell within the Overlap zone and 44 species (69.8%) within the Innovation zone, while three species (4.8%) were classified as De-prioritised. No species met the criteria for the Gap zone. The De-prioritised taxa were *Aristolochia assamica*, *Rubus moluccanus*, and *Phlogacanthus tubiflorus* (Fig. 6). *Artocarpus heterophyllus* was assigned to evidence tier E = 2 and placed in the Innovation zone because both in vivo and in vitro antidiabetic evidence were identified. Only two taxa were assigned to E = 1 (in vitro evidence only): *Hydrocotyle sibthorpioides* within the high-salience group and *Alocasia odora* within the Innovation group. Fidelity Level values for diabetes-specific use are reported in Table 2.

**Table 2:** Taxonomic classification, growth forms, utilized plant parts, traditional preparation modes, Relative Frequency of Citation (RFC), Use Value (UV), and Fidelity Level (FL) of antidiabetic plant species documented in Biswanath district, Assam, India.

Botanical name (Voucher No.)	Vernacular name	Family	Habit	Parts used	Mode of preparation for diabetes	FC for diabetes	RFC for diabetes	Use category	∑Ui (total use-reports across all ailments)	UV (overall Use Value across all ailments)	Nu (informants citing the species for any ailment)	Np (specific-ailment informants)	FL (%)	GPS (°*N*, °E)
*Aegle marmelos* (L.) Corrêa (HAU/AN-3780)	Bel	Rutaceae	Tree	Fruit, leaves	Ripe fruit eaten raw or as juice; leaves boiled in water	62	0.141	4	281	0.639	108	Diabetes (62)	57.41	26.8305, 93.6869
												Constipation (41)	37.96	
												Diarrhoea (86)	79.63	
												Dysentery (92)	85.19	
*Allium sativum* L. (HAU/AN-3781)	Nohoru	Amaryllidaceae	Herb	Leaf, bulb	Bulb juice	58	0.132	6	1038	2.359	261	Diabetes (58)	22.22	26.7283, 93.0532
												Asthma (146)	55.94	
												Body pain (245)	93.87	
												Cold & fever (256)	98.08	
												Hypertension (155)	59.39	
												Rheumatism (178)	68.20	
*Alocasia odora* (G.Lodd.) Spach (HAU/AN-3782)	Dahi kosu	Araceae	Shrub	Young petiole	Petioles boiled and eaten	10	0.023	2	23	0.052	21	Diabetes (10)	47.62	26.8332, 93.2645
												Constipation (13)	61.91	
*Alpinia galanga* (L.) Willd. (HAU/AN-3783)	Karphul	Zingiberaceae	Herb	Rhizome	Rhizome chewed on empty stomach	20	0.045	4	241	0.548	160	Diabetes (20)	12.50	26.8762, 93.2726
												Cold & fever (51)	31.88	
												Diarrhoea (35)	21.88	
												Rheumatism (135)	84.38	
*Alstonia scholaris* (L.) R.Br. (HAU/AN-3784)	Sotiana	Apocynaceae	Tree	Bark	Bark taken on empty stomach	42	0.095	3	174	0.395	97	Diabetes (42)	43.30	26.8202, 93.4026
												Malaria (65)	67.01	
												Pneumonia (67)	69.07	
*Alternanthera sessilis* (L.) DC. (HAU/AN-3785)	Matikaduri	Amaranthaceae	Herb	Young shoot	Juice of young shoots	18	0.041	3	69	0.157	47	Diabetes (18)	38.30	26.8693, 93.0932
												Dysentery (34)	72.34	
												Skin diseases (17)	36.17	
*Amaranthus spinosus* L. (HAU/AN-3786)	Hati-khutura	Amaranthaceae	Herb	Root, young shoot	Juice; cooked as vegetable	52	0.118	2	88	0.2	71	Diabetes (52)	73.24	26.7881, 93.4196
												Dysentery (36)	50.70	
*Andrographis paniculata* (Burm.f.) Wall. ex Nees (HAU/AN-3787)	Chirata	Acanthaceae	Herb	Leaf	Dried leaves infused in warm water	98	0.223	6	460	1.045	132	Diabetes (98)	74.24	26.8411, 93.4112
												Chicken pox (52)	39.39	
												Cold & fever (78)	59.10	
												Inflammatory Bowel Disease (55)	41.68	
												Malaria (130)	98.49	
												Pneumonia (47)	35.61	
*Aristolochia assamica* D.Borah & T.V.Do (HAU/AN-3788)	Nilakantha	Aristolochiaceae	Vine	Leaf, root	Juice; infusion	42	0.095	4	108	0.245	57	Diabetes (42)	73.68	26.8534, 93.7021
												Malaria (11)	19.30	
												Pneumonia (34)	59.65	
												Rheumatism (21)	36.84	
*Artocarpus heterophyllus* Lam. (HAU/AN-3789)	Kothal	Moraceae	Tree	Seed	Boiled seeds eaten	6	0.014	2	25	0.057	24	Diabetes (6)	25	26.8553, 93.0853
												Constipation (19)	79.17	
*Asparagus racemosus* Willd. (HAU/AN-3790)	Satmul	Asparagaceae	Herb	Tuber	Tuber juice mixed with water or milk	38	0.086	5	280	0.636	95	Diabetes (38)	40	26.8381, 93.7151
												Diarrhoea (61)	64.21	
												Epilepsi (17)	17.89	
												Rheumatism (81)	85.26	
												Uterine problem (83)	87.37	
*Averrhoa carambola* L. (HAU/AN-3791)	Kordoi	Oxalidaceae	Tree	Fruit	Fruit cooked as curry	10	0.023	2	38	0.086	31	Diabetes (10)	32.26	26.8708, 93.3389
												Dysentery (28)	90.32	
*Azadirachta indica* A.Juss. (HAU/AN-3792)	Mohaneem	Meliaceae	Tree	Leaf	Leaf juice	69	0.157	8	605	1.375	205	Diabetes (69)	33.66	26.8495, 93.7015
												Asthma (48)	23.42	
												Chicken pox (171)	83.42	
												Jaundice (56)	27.32	
												Malaria (74)	36.10	
												Pneumonia (11)	5.37	
												Rheumatism (86)	41.95	
												Skin diseases (90)	43.90	
*Bombax ceiba* L. (HAU/AN-3793)	Himolu	Malvaceae	Tree	Bark	Bark juice	16	0.036	3	91	0.207	53	Diabetes (16)	30.19	26.8549, 93.6476
												Jaundice (34)	64.15	
												Uterine problem (41)	77.36	
*Catharanthus roseus* (L.) G.Don (HAU/AN-3794)	Nayantara	Apocynaceae	Herb	Leaf, flower, root	Flowers/leaves crushed to obtain juice	24	0.055	4	134	0.305	68	Diabetes (24)	35.29	26.8290, 93.1013
												Cancer (22)	32.35	
												Hypertension (45)	66.18	
												Malaria (43)	63.24	
*Centella asiatica* (L.) Urb. (HAU/AN-3795)	Bor manimuni	Apiaceae	Herb	Leaf	Leaves chewed on empty stomach	60	0.136	4	175	0.398	135	Diabetes (60)	44.44	26.9729, 93.6802
												Cuts and wound healing (21)	15.56	
												Dysentery (68)	50.37	
												Malaria (26)	19.26	
*Clerodendrum infortunatum* L. (HAU/AN-3796)	Dhopat teeta	Lamiaceae	Shrub	Root	Root juice	30	0.068	4	140	0.318	74	Diabetes (30)	40.54	26.8176, 93.1691
												Asthma (39)	52.70	
												Malaria (28)	37.84	
												Rheumatism (43)	58.19	
*Coccinia grandis* (L.) Voigt (HAU/AN-3797)	Kunduli	Cucurbitaceae	Vine	Root	Root juice	8	0.018	2	16	0.036	15	Diabetes (8)	53.33	26.8500, 93.3300
												Jaundice (8)	53.33	
*Curcuma longa* L. (HAU/AN-3798)	Halodhi	Zingiberaceae	Herb	Rhizome	Dried rhizome boiled in water	34	0.077	6	987	2.243	345	Diabetes (34)	9.86	26.9320, 93.5406
												Body pain (243)	70.43	
												Cold and fever (281)	81.45	
												Cuts and wound healing (250)	72.46	
												Indigestion (55)	15.94	
												Rheumatism (124)	35.94	
*Cuscuta reflexa* Roxb. (HAU/AN-3799)	Akakhilota	Convolvulaceae	Vine	Whole plant	Raw paste; decoction/juice also used	36	0.082	2	68	0.155	47	Diabetes (36)	76.6	26.9415, 93.7266
												Swelling (32)	68.09	
*Dillenia indica* L. (HAU/AN-3800)	Ou tenga	Dilleniaceae	Tree	Fruit	Decoction; juice; infusion	54	0.123	3	188	0.427	81	Diabetes (54)	66.67	26.7552, 93.2946
												Constipation (64)	79.01	
												Dysentery (70)	86.42	
*Elaeagnus angustifolia* L. (HAU/AN-3801)	Mirika	Elaeagnaceae	Shrub	Fruit	Raw fruit eaten	12	0.027	4	83	0.189	33	Diabetes (12)	36.36	26.8090, 93.3216
												Diarrhoea (28)	84.85	
												Dysentery (20)	60.61	
												Indigestion (23)	69.70	
*Eleusine indica* (L.) Gaertn. (HAU/AN-3802)	Bobosa bon	Poaceae	Herb	Whole plant	Young seedlings eaten raw	4	0.009	2	13	0.03	12	Diabetes (4)	33.33	26.8187, 93.0903
												Dysentery (9)	75	
*Ficus racemosa* L. (HAU/AN-3803)	Mau dimoru	Moraceae	Tree	Fruit	Boiled fruit eaten	10	0.023	3	63	0.143	37	Diabetes (10)	27.03	26.8043, 93.1846
												Constipation (31)	83.78	
												Diarrhoea (22)	59.46	
*Ficus religiosa* L. (HAU/AN-3804)	Ahot	Moraceae	Tree	Leaf, bark	Dry bark powder mixed with water	8	0.018	2	22	0.05	19	Diabetes (8)	42.11	26.9436, 93.5435
												Diarrhoea (14)	73.68	
*Garcinia lanceifolia* Roxb. (HAU/AN-3805)	Rupohi thekera	Clusiaceae	Shrub	Fruit	Dried fruit soaked in warm water; raw	18	0.041	2	124	0.282	120	Diabetes (18)	15	26.8531, 93.0767
												Dysentery (106)	83.33	
*Garcinia pedunculata* Roxb. ex Buch.-Ham. (HAU/AN-3806)	Bor thekera	Clusiaceae	Tree	Fruit	Dried fruit soaked in warm water; raw	38	0.086	4	411	0.934	211	Diabetes (38)	18.01	26.8424, 93.6983
												Diarrhoea (151)	71.56	
												Dysentery (167)	79.15	
												Inflammatory Bowel Disease (55)	26.07	
*Garcinia xanthochymus* Hook.f. ex T.Anderson (HAU/AN-3807)	Tepor tenga	Clusiaceae	Tree	Fruit	Dried fruit soaked in warm water; raw	24	0.055	4	433	0.984	178	Diabetes (24)	13.48	26.9450, 93.7134
												Diarrhoea (171)	96.07	
												Dysentery (159)	89.33	
												Inflammatory Bowel Disease (79)	44.39	
*Guilandina bonduc* L. (Syn. *Caesalpinia bonduc* (L.) Roxb.) (HAU/AN-3808)	Letaguti	Fabaceae	Tree	Seed	Seed juice	46	0.105	3	210	0.477	91	Diabetes (46)	50.55	26.8732, 93.2861
												Cold and fever (87)	95.60	
												Pneumonia (77)	84.62	
*Hellenia speciosa* (J.Koenig) S.R.Dutta (HAU/AN-3809)	Jom lakhuti	Costaceae	Shrub	Stem, rhizome, leaf	Juice; infusion	22	0.050	3	148	0.336	112	Diabetes (22)	19.64	26.8576, 93.7180
												Asthma (75)	66.96	
												Cuts and wound healing (51)	45.54	
*Hydrocotyle sibthorpioides* Lam. (HAU/AN-3810)	Horu manimuni	Araliaceae	Herb	Whole plant	Decoction; juice	63	0.143	4	213	0.484	135	Diabetes (63)	46.67	26.7923, 93.4093
												Body pain (42)	31.11	
												Cuts and wound healing (67)	49.63	
												Dysentery (41)	30.37	
*Ipomoea aquatica* Forssk. (HAU/AN-3811)	Pani kolmow	Convolvulaceae	Herb	Tender shoot	Boiled tender shoots eaten	30	0.068	2	51	0.116	37	Diabetes (30)	81.08	26.8450, 93.0836
												Cuts and wound healing (21)	56.76	
*Kalanchoe pinnata* (Lam.) Pers. (HAU/AN-3812)	Dupar tenga	Crassulaceae	Herb	Leaf	Fresh leaf juice on empty stomach	88	0.200	3	155	0.352	101	Diabetes (88)	87.13	26.8750, 93.2959
												Cuts and wound healing (21)	20.79	
												Dysentery (46)	45.55	
*Leucas aspera* (Willd.) Link (HAU/AN-3813)	Durun	Lamiaceae	Herb	Young shoot	Young shoots cooked as meal	56	0.127	6	252	0.573	146	Diabetes (56)	38.36	26.8241, 93.1466
												Body pain (78)	53.42	
												Dysentery (49)	33.56	
												Pneumonia (11)	7.53	
												Rheumatism (25)	17.12	
												Skin diseases (33)	22.60	
*Momordica charantia* L. (HAU/AN-3814)	Teeta kerela	Cucurbitaceae	Vine	Fruit, leaf	Juice	40	0.091	4	122	0.277	114	Diabetes (40)	35.09	26.8218, 93.2467
												Cuts and wound healing (23)	20.18	
												Malaria (53)	46.49	
												Skin diseases (6)	5.26	
*Moringa oleifera* Lam. (HAU/AN-3815)	Sojina	Moringaceae	Tree	Seed, fruit	Pods cooked; seed powder taken with warm water	24	0.055	4	215	0.489	98	Diabetes (24)	24.49	26.9490, 93.7083
												Diarrhoea (67)	68.37	
												Hypertension (54)	55.10	
												Rheumatism (70)	71.43	
*Morus alba* L. (HAU/AN-3816)	Nuni	Moraceae	Tree	Fruit, leaf	Raw fruit; leaf juice	36	0.082	2	72	0.164	49	Diabetes (36)	73.47	26.9471, 93.7017
												Hypertension (36)	73.47	
*Murraya koenigii* (L.) Spreng. (syn. of *Bergera koenigii* L.) (HAU/AN-3817)	Narasingha	Rutaceae	Shrub	Leaf, flower, bark	Leaves chewed on empty stomach	14	0.032	3	219	0.498	183	Diabetes (14)	7.65	26.8604, 93.2808
												Body pain (158)	86.34	
												Cold and fever (47)	25.63	
*Musa balbisiana* Colla (HAU/AN-3818)	Athiya kol	Musaceae	Herb	Stem	Paste; juice	22	0.050	4	112	0.255	64	Diabetes (22)	34.38	26.7980, 93.1471
												Diarrhoea (32)	50	
												Dysentery (24)	37.5	
												Pneumonia (34)	53.13	
*Nyctanthes arbor-tristis* L. (HAU/AN-3819)	Sewali	Oleaceae	Shrub	Flower	Infusion	79	0.180	5	436	0.991	202	Diabetes (79)	39.11	26.8093, 93.1413
												Body pain (87)	43.07	
												Cold and fever (176)	87.13	
												Diarrhoea (37)	18.32	
												Malaria (57)	28.22	
*Ocimum tenuiflorum* L. (HAU/AN-3820)	Tulsi	Lamiaceae	Herb	Leaf	Decoction; juice	36	0.082	9	866	1.968	312	Diabetes (36)	11.54	26.8358, 93.5991
												Asthma (141)	45.19	
												Body pain (154)	49.36	
												Cold and fever (270)	86.54	
												Diarrhoea (23)	7.37	
												Dysentery (57)	18.27	
												Jaundice (30)	9.62	
												Malaria (58)	18.59	
												Pneumonia (97)	31.09	
*Oldenlandia corymbosa* L. (HAU/AN-3821)	Senibon	Rubiaceae	Herb	Whole plant	Juice	36	0.082	3	88	0.2	54	Diabetes (36)	66.67	26.8500, 93.3301
												Jaundice (39)	72.22	
												Pneumonia (13)	24.07	
*Oroxylum indicum* (L.) Kurz (HAU/AN-3822)	Bhat ghila	Bignoniaceae	Tree	Root bark, seed bark, leaf	Infusion	26	0.059	5	225	0.511	78	Diabetes (26)	33.33	26.7096, 93.1395
												Jaundice (27)	34.62	
												Rheumatism (67)	85.90	
												Swelling (51)	65.39	
												Uterine problem (54)	69.23	
*Oxalis corniculata* L. (HAU/AN-3823)	Tengesi	Oxalidaceae	Herb	Whole plant	Juice	44	0.100	3	129	0.293	68	Diabetes (44)	64.71	26.8388, 93.2344
												Cuts and wound healing (24)	35.29	
												Dysentery (61)	89.71	
*Phlogacanthus tubiflorus* Nees (HAU/AN-3824)	Ranga bahak; Titaphul	Acanthaceae	Shrub	Flower	Flowers cooked as meal	6	0.014	4	122	0.277	96	Diabetes (6)	6.25	26.8381, 93.7148
												Cold and fever (57)	59.38	
												Pneumonia (34)	35.42	
												Skin diseases (25)	26.04	
*Phyllanthus emblica* L. (HAU/AN-3825)	Amlakhi	Phyllanthaceae	Tree	Fruit	Infusion	58	0.132	6	922	2.095	330	Diabetes (58)	17.58	26.8707, 93.3390
												Constipation (82)	24.85	
												Diarrhoea (189)	57.27	
												Dysentery (209)	63.33	
												Indigestion (288)	87.23	
												Inflammatory Bowel Disease (96)	29.09	
*Physalis angulata* L. (HAU/AN-3826)	Phutkala	Solanaceae	Herb	Root	Infusion; juice	22	0.050	2	53	0.12	37	Diabetes (22)	59.46	26.7889, 93.4192
												Indigestion (31)	83.78	
*Piper longum* L. (HAU/AN-3827)	Pipoli	Piperaceae	Vine	Fruit, root	Decoction	60	0.136	3	146	0.332	93	Diabetes (60)	64.52	26.8547, 93.6450
												Asthma (49)	52.69	
												Pneumonia (37)	39.78	
*Psidium guajava* L. (HAU/AN-3828)	Madhuri	Myrtaceae	Tree	Young shoot	Juice	38	0.086	4	285	0.648	163	Diabetes (38)	23.31	26.8341, 93.6052
												Diarrhoea (85)	52.15	
												Dysentery (115)	70.55	
												Inflammatory Bowel Disease (47)	28.83	
*Rubus moluccanus* L. (HAU/AN-3829)	Jetulipoka	Rosaceae	Shrub	Root, young shoot	Decoction	28	0.064	2	42	0.095	40	Diabetes (28)	70	26.9734, 93.6826
												Malaria (14)	35	
*Scoparia dulcis* L. (HAU/AN-3830)	Bonjaluk	Plantaginaceae	Herb	Whole plant	Decoction	26	0.059	2	47	0.107	29	Diabetes (26)	89.66	26.9433, 93.7026
												Hypertension (21)	72.41	
*Solanum nigrum* L. (HAU/AN-3831)	Loskochi	Solanaceae	Herb	Tender shoot	Tender shoots cooked and eaten	23	0.052	2	52	0.118	48	Diabetes (23)	47.92	26.8499, 93.3301
												Dysentery (29)	60.42	
*Solanum violaceum* Ortega (HAU/AN-3832)	Tita bhekuri	Solanaceae	Shrub	Fruit	Fruit eaten raw or boiled	54	0.123	2	89	0.202	80	Diabetes (54)	67.5	26.9402, 93.5367
												Malaria (35)	43.75	
*Spondias pinnata* (L.f.) Kurz (HAU/AN-3833)	Omora	Anacardiaceae	Tree	Fruit, tender leaves	Eaten raw	42	0.095	2	105	0.239	91	Diabetes (42)	46.15	26.8523, 93.0916
												Dysentery (63)	69.23	
*Syzygium aromaticum* (L.) Merr. & L.M.Perry (HAU/AN-3834)	Laung	Myrtaceae	Tree	Flower bud	Infusion	30	0.068	4	400	0.909	217	Diabetes (30)	13.82	26.7551, 93.2951
												Asthma (94)	43.32	
												Body pain (75)	34.56	
												Cold and fever (201)	92.63	
*Syzygium cumini* (L.) Skeels (HAU/AN-3835)	Kolajamu	Myrtaceae	Tree	Fruit	Raw fruit eaten	75	0.170	2	97	0.22	81	Diabetes (75)	92.59	26.8089, 93.3215
												Dysentery (22)	27.16	
*Syzygium jambos* (L.) Alston (HAU/AN-3836)	Bogajamu	Myrtaceae	Tree	Tender leaves, fruits	Leaves and fruits used (local use reports); often eaten raw	6	0.014	2	24	0.055	24	Diabetes (6)	25	26.8479, 93.0854
												Indigestion (18)	75	
*Terminalia arjuna* (Roxb. ex DC.) Wight & Arn. (HAU/AN-3837)	Arjun	Combretaceae	Tree	Bark	Bark decoction (strained); powdered bark mixed with warm water	20	0.045	5	156	0.355	98	Diabetes (20)	20.41	26.9518, 93.7021
												Asthma (27)	27.55	
												Cuts and wound healing (34)	34.69	
												Hypertension (58)	59.18	
												Pneumonia (17)	17.35	
*Terminalia bellirica* (Gaertn.) Roxb. (HAU/AN-3838)	Bhumura	Combretaceae	Tree	Fruit	Dried fruit powder mixed with water; often with *T. chebula* and *P. emblica*	18	0.041	4	515	1.17	201	Diabetes (18)	8.96	26.8267, 93.6010
												Diarrhoea (197)	98.01	
												Dysentery (191)	95.03	
												Indigestion (109)	54.23	
*Terminalia chebula* Retz. (HAU/AN-3839)	Silikha	Combretaceae	Tree	Fruit	Dried fruit chewed	32	0.073	5	805	1.83	240	Diabetes (32)	13.33	26.7008, 93.1399
												Constipation (161)	67.08	
												Diarrhoea (204)	85	
												Dysentery (191)	79.58	
												Indigestion (217)	90.42	
*Tinospora cordifolia* (Willd.) Hook.f. & Thomson (HAU/AN-3840)	Amarlota; Sogunilota	Menispermaceae	Vine	Stem	Boiled stem eaten on empty stomach	40	0.091	4	253	0.575	217	Diabetes (40)	18.43	26.8594, 93.2222
												Diarrhoea (36)	16.59	
												Rheumatism (45)	20.74	
												Swelling (132)	60.83	
*Trigonella foenum-graecum* L. (HAU/AN-3841)	Methi guti	Fabaceae	Herb	Seed	Seeds soaked overnight; taken on empty stomach	52	0.118	3	167	0.38	70	Diabetes (52)	74.29	26.8499, 93.3299
												Constipation (57)	81.43	
												Indigestion (58)	82.86	
*Vitex negundo* L. (HAU/AN-3842)	Posotia	Lamiaceae	Shrub	Leaf	Leaves soaked in warm water and drunk on empty stomach	35	0.080	4	217	0.493	75	Diabetes (35)	46.67	26.7571, 93.2962
												Cold and fever (56)	74.67	
												Pneumonia (67)	89.33	
												Rheumatism (59)	78.68	

## Discussion

This study documents medicinal plants used for diabetes-related complaints in Biswanath district, Assam, based on ethnobotanical knowledge recorded from 440 informants.

In total, 63 plant species representing 57 genera and 39 families were identified (Tables 1 and 2). The diversity of taxa reported reflects an active tradition of plant-based healthcare embedded within the district’s heterogeneous agro-ecological landscapes (Figs. 1, 2 and 3)^13^. In terms of growth form, trees constituted the largest proportion of recorded taxa (38%), followed by herbs (35%), shrubs (17%) and vines (10%) (Fig. 4). Comparable patterns are frequently observed in ethnobotanical inventories where perennial species remain accessible throughout the year and provide multiple harvestable plant parts for household medicinal use^37^.

Regarding plant parts utilised, fruit (22.9%) and leaf (21.7%) were the most frequently reported categories, followed by root (10.8%) and shoot (8.4%), whereas bark accounted for 6.0% of the 83 plant-part use records (Fig. 5d). The predominance of fruits and leaves is notable because these parts can often be harvested with comparatively lower ecological impact than bark or roots, although sustainability ultimately depends on local abundance and harvesting intensity^13,14^. The species–part matrix indicates that most taxa were associated with one or two principal plant parts, whereas a smaller subset was reported across multiple parts (Fig. 5c). Among the individually specified preparation methods, juice was the most frequently recorded (24 species), while powder and infusion were each recorded for four species, decoction for three species, and paste for one species; the remaining 27 species were grouped under “Other” preparation or consumption modes (Fig. 5e). Differences in preparation methods may influence exposure, dosage consistency, and bioavailability of plant constituents, underscoring the importance of clearer reporting and improved standardisation in future pharmacological investigations^26^.

Relative Frequency of Citation (RFC) identified a concentrated group of culturally salient taxa, led by *Andrographis paniculata* (RFC = 0.223), followed by *Kalanchoe pinnata* (0.200), *Nyctanthes arbor-tristis* (0.180), *Syzygium cumini* (0.170), and *Azadirachta indica* (0.157) (Fig. 5a–b; Table 2). These frequently cited species represent practical entry points for follow-up studies focusing on reproducible preparation practices, safety documentation, and quality standardisation^28^. Correlation analysis among plant-part use, preparation modes, and citation frequency did not indicate a strong association between RFC and part-use diversity (Fig. 5f). Instead, the correlation structure highlights broader patterns of plant-part utilisation and preparation practices within the local ethnomedicinal system. Complementary ethnobotanical indices provide additional insight into species importance (Table 2): Use Value (UV) reflects overall ethnomedicinal versatility across all recorded ailments and should not be interpreted as a measure of antidiabetic importance, whereas RFC and FL provide diabetes-specific measures of cultural salience and informant consensus, respectively^16,37^. Although a higher FL indicates stronger diabetes-specific consensus, a comparatively low FL in a multipurpose species may result from a larger denominator (Nu) because the species is cited for several ailments, while the number of diabetes-specific citations (Np) remains unchanged; therefore, a low FL should not be interpreted as indicating weak relevance to the reported diabetes-related use, and FL should not be regarded as a measure of pharmacological efficacy.

An important analytical component of this study is the integration of ethnobotanical salience with published pharmacological evidence using the RFC × evidence-tier framework (Fig. 6)^10,28^. Within this framework, 16 species were positioned in the Overlap zone (high salience; E ≥ 1), indicating taxa that are both culturally prominent and supported by experimental antidiabetic evidence. Most species occurred within the Innovation zone (lower salience; E ≥ 1), suggesting that published evidence is available for several taxa cited less frequently in the present ethnobotanical survey.

Importantly, no highly cited taxon met both Gap-zone criteria: high cultural salience (RFC ≥ P75) and absence of published antidiabetic evidence (E = 0). Consequently, no species was placed in the Gap zone under the applied search and eligibility criteria. Because evidence was graded at the species level, this result indicates that all highly salient taxa had at least one published antidiabetic study identified in the literature search. However, it should be interpreted within the scope of the literature search and does not constitute experimental validation of the specific plant parts or preparations documented locally^26,28^.

Within this prioritisation, two taxa—*Hydrocotyle sibthorpioides* and *Alocasia odora*—were supported only by in vitro evidence (E = 1) and therefore represent candidates for strengthening the evidence base through well-designed in vivo studies and improved phytochemical characterisation (Fig. 6; Table 3). Conversely, three taxa—*Aristolochia assamica*, *Rubus moluccanus*, and *Phlogacanthus tubiflorus*—were positioned in the lower-salience, no-eligible-evidence zone. Their placement reflects the absence of eligible direct antidiabetic evidence under the applied search criteria rather than a lack of ethnomedicinal relevance. *Artocarpus heterophyllus*, for which both in vivo and in vitro antidiabetic evidence were identified, was assigned to E = 2 and placed in the Innovation zone. Although *A. assamica* was reported in active local use, its documentation in this study should not be interpreted as an endorsement, recommendation, or confirmation of safety. Given the well-established nephrotoxicity and carcinogenicity associated with aristolochic acids found in members of the genus *Aristolochia*, the medicinal use of this taxon raises serious safety concerns and requires species-, plant-part-, preparation-, and dose-specific toxicological evaluation.

**Table 3:** Summary of in vivo and in vitro studies on antidiabetic plants reported from Biswanath district, Assam, India.

Plant name	In vivo anti-diabetic model	Effect/s	In vitro anti-diabetic model	Effect/s
*Aegle marmelos* (L.) Corrêa	STZ induced diabetic Wistar rats	Increased level of pancreatic insulin secretion^40,41^	Enzyme assay	Alpha-amylase and alpha-glucosidase inhibition^42,43^
*Allium sativum* L.	STZ and Alloxan induced diabetic Sprague-Dawley rats	Antihyperglycemic, reduced serum glucose, cholesterol and triglyceride levels^44,45^	Enzyme assay	Inhibition of hepatic glucose production, alpha-amylase alpha-glucosidase inhibition, increased glucose uptake e by the L-6 cell lines^46^
*Alocasia odora* (G.Lodd.) Spach			Enzyme assay	Alpha-glucosidase inhibition^47^
*Alpinia galanga* (L.) Willd.	STZ induced diabetic Wistar Albino rats	Β-cells regeneration^48^	Enzyme assay	Alpha-glucosidase and alpha-amylase inhibition^49,50^
*Alstonia scholaris* (L.) R.Br.	STZ induced diabetic Wistar Albino rats, normoglycemic Albino mice	Antihyperglycemic, antihyperlipidemic hypoglycemic^51,52^	Enzyme assay	Alpha-glucosidase inhibition^53^
*Alternanthera sessilis* (L.) DC.	High fat diet and STZ induced Sprague–Dawely rats, STZ induced Wistar rats, Normal mice	Reduced blood glucose, improved insulin sensitivity, antihyperglycemic, hypoglycemic, antihyperlipidemic^54,55^	Enzyme assay	Alpha-glucosidase inhibition^56^
*Amaranthus spinosus* L.	STZ and Alloxan induced diabetic Albino rats	Decreased plasma glucose, hepatic glucose-6-phophatase activity, Levels of lipids in both plasma and liver, along with urea, creatinine, and markers of lipid peroxidation, were assessed, increased hepatic glycogen content^57,58^	Enzyme assay	Alpha-amylase inhibition^59^
*Andrographis paniculata* (Burm.f.) Wall. ex Nees	High fat diet and STZ induced diabetic Sprague–Dawley rats,	Improved metabolic profile, reduced fasting blood glucose, moderately improved pancreatic β-cells^60,61^	Enzyme assay	Alpha-glucosidase and alpha-amylase inhibition^62^
*Aristolochia assamica* D.Borah & T.V.Do				
*Artocarpus heterophyllus* Lam.	STZ induced diabetic Wistar rats	Reduced serum glucose, cholesterol and triglyceride levels, antihyperglycemia, antihyperlipidemia^63–65^	Enzyme assay	Alpha-glucosidase and alpha-amylase inhibition^65^
Asparagus racemosus Willd.	STZ induced diabetic Wistar rats	Hypoglycemic, hypolipidemic activity^66^	Enzyme assay	Alpha-glucosidase and alpha-amylase inhibition^66,67^
*Averrhoa carambola* L.	STZ induced diabetic mice	Reduced serum glucose, total cholesterol, triglyceride, free fatty acids, increased insulin^68^	Enzyme assay	Alpha-glucosidase inhibition^69^
*Azadirachta indica* A.Juss.	STZ induced diabetic BALB/C mice, Alloxan induced diabetic Swiss Albino mice	Reduced blood glucose level, pancreatic and liver cell regeneration, hypoglycemic, antihyperglycemic^70–72^	Enzyme assay	Increased glucose uptake by yeast cells, alpha-amylase inhibition, glucose adsorption^72^
*Bombax ceiba* L.	Alloxan induced diabetic Swiss Webster mice, Normal Swiss Albino mice	Hypoglycemic, hypolipidemic and hepato protective^73,74^	Enzyme assay	Alpha-glucosidase and alpha-amylase inhibition^75,76^
*Catharanthus roseus* (L.) G.Don	STZ induced diabetic Sprague–Dawley rats and Wistar rats	Enhanched expression of GLUT gene, hypoglycemic^77,78^	Enzyme assay	Alpha-glucosidase and alpha-amylase inhibition^79^
*Centella asiatica* (L.) Urb.	Lipid emulsion-induced hyperlipidemic rat, STZ induced diabetic Long Evan rats	Hypolipidemic and hypoglycemic, anti-hyperglycemic^80,81^	Enzyme assay	Alpha-glucosidase inhibition^82^
*Clerodendrum infortunatum* L.	STZ induced diabetic Long-Evans rats and Wistar rats	Reduced serum glucose, total cholesterol, and low-density lipoprotein level, increased while high-density lipoprotein level, anti-hyperglycemic^83,84^	Enzyme assay	Alpha-amylase and alpha-glucosidase inhibition^83,85^
*Coccinia grandis* (L.) Voigt	STZ induced diabetic Wistar Albino rat	Reduced blood glucose, increased body weight and serum insulin^86^	Enzyme assay	Alpha-amylase and alpha-glucosidase inhibition^87,88^
*Curcuma longa* L.	Alloxan induced diabetic mice	Reduced fasting blood glucose level^89^	Enzyme assay	Alpha-amylase, alpha-glucosidase and beta-glucosidase inhibition^90,91^
*Cuscuta reflexa* Roxb.	STZ induced diabetic Swiss Albino rats, Alloxan induced diabetic Wistar Albino rats	Reduction of blood glucose level, decreased hba1c and improved insulin levels, improvement in liver function test and lipid profile test^92,93^	Enzyme assay	Alpha-amylase inhibition^94^
*Dillenia indica* L.	STZ induced diabetic Wistar rats	Advanced glycation end products inhibitory activity in kidneys^95^	Enzyme assay	Alpha-amylase and alpha-glucosidase inhibition^96^
*Elaeagnus angustifolia* L.	STZ induced diabetic Sprague–Dawley rats	Reduced blood glucose level, improved glucose tolerance and enhanced body weight^97^	Enzyme assay	Alpha-amylase and alpha-glucosidase inhibition^98^
*Eleusine indica* (L.) Gaertn.	Alloxan-induced diabetic rats	Significant reduction in fasting blood glucose levels^99^		
*Ficus racemosa* L.	High-fat meal- and STZ-induced hypercholesterolaemia-associated diabetic rats	Reduced blood glucose, total cholesterol, triglycerides, and LDL levels, and restored serum insulin and HDL levels^100^	Enzyme assay	Significant dose-dependent inhibition of α-amylase and α-glucosidase^101,102^
*Ficus religiosa* L.	Streptozotocin (STZ)-induced diabetic rats; alloxan-induced diabetic rats.	Reduced blood glucose, serum triglyceride, and total cholesterol levels, with increased serum insulin, body weight, and glycogen content in the liver and skeletal muscle^103^. Reduced blood glucose and improved serum lipid profile, including reductions in serum cholesterol and triglycerides^104^	α-Amylase and α-glucosidase inhibition assays	α-Amylase and α-glucosidase inhibition^105,106^
*Garcinia lanceifolia* Roxb.	STZ–nicotinamide-induced diabetic Wistar albino rats; oral glucose tolerance test in Swiss-albino mice	Significant antihyperglycaemic activity^107,108^	α-Amylase and α-glucosidase inhibition assays	Concentration-dependent inhibition of α-amylase and α-glucosidase^109^
*Garcinia pedunculata* Roxb. ex Buch.-Ham.	STZ induced diabetic Wistar Albino rats, high fat diet induced wister Albino rat	Reduction in the body weight, serum total cholesterol, triglycerides, LDL and liver biomarker enzymes^110,111^	Enzyme assay	Alpha-amylase and alpha-glucosidase inhibition^111,112^
*Garcinia xanthochymus* Hook.f. ex T.Anderson	Alloxan-induced diabetic mice	Reduced blood glucose levels and improved serum biochemical/lipid parameters^113^.	α-Amylase and α-glucosidase inhibition assays; glucose uptake assay	α-Amylase and α-glucosidase inhibition and increased glucose uptake^114^
*Guilandina bonduc* L. (Syn. *Caesalpinia bonduc* (L.) Roxb.)	Alloxan induced diabetic Wistar Albino rat, STZ induced diabetic Wistar Albino rat	Reduced blood glucose level, total cholesterol, triglyceride, lowered blood urea nitrogen level, antihyperglycemic, antihyperlipidemic^115–117^	α-Amylase and α-glucosidase inhibition assays; glucose adsorption and diffusion assays; in vitro amylolysis kinetics	α-Amylase and α-glucosidase inhibition, increased glucose adsorption capacity, inhibition of glucose diffusion, and increased glucose uptake^118,119^.
*Hellenia speciosa* (J.Koenig) S.R.Dutta	Alloxan induced diabetic rats, STZ induced diabetic Wistar rat	Reduced plasma glucose, decreased glycosylated hemoglobin (hba1c), serum total cholesterol, triglyceride, LDL cholesterol, increased plasma insulin, tissue glycogen, HDL cholesterol and serum protein, increase in glucokinase, aldolase, pyruvate kinase, succinate dehydrogenase, and glycogen synthase activities^120–122^	Enzyme assay	Alpha-amylase and alpha-glucosidase inhibition^123,124^
*Hydrocotyle sibthorpioides* Lam.			Enzyme assay	Alpha-amylase and alpha-glucosidase inhibition^125^
*Ipomoea aquatica* Forssk.	STZ induced diabetic Wistar rats	Reduced fasting blood glucose levels^126^.	α-Glucosidase inhibition assay	α-Glucosidase inhibition^127^.
*Kalanchoe pinnata* (Lam.) Pers.	STZ-induced diabetic male albino Wistar rats	Reduced fasting blood glucose levels, improved lipid profile, and increased insulin secretion^128^	α-Amylase and α-glucosidase inhibition assays	α-Amylase and α-glucosidase inhibition^129,130^.
*Leucas aspera* (Willd.) Link	STZ-induced diabetic Wistar albino rats	Reduced blood glucose, total cholesterol and triglyceride levels^131^.	C2C12 cell-line glucose-uptake assay; α-amylase and α-glucosidase inhibition assays	Enhanced glucose uptake; α-amylase and α-glucosidase inhibition by *L. aspera*-mediated CuNPs^132,133^
*Momordica charantia* L.	Alloxan-induced diabetic rats and mice; STZ–nicotinamide-induced diabetic Wistar rats; high-fat-diet + STZ-induced diabetic Wistar rats	Reduced blood glucose and improved glucose tolerance; reduced serum total cholesterol, triglycerides and non-esterified fatty acids; increased insulin content and insulin sensitivity; and reduced serum urea, creatinine, ALT, AST and ALP levels^134–137^.	α-Amylase and α-glucosidase inhibition assays	α-Amylase and α-glucosidase inhibition^136,138^.
*Moringa oleifera* Lam.	Alloxan-induced diabetic Wistar rats; alloxan-induced diabetic mice	Reduced blood glucose, total cholesterol and triglyceride levels; reduced insulin resistance and improved renal biochemical parameters, including creatinine and blood urea nitrogen^139–141^.	α-Amylase and α-glucosidase inhibition assays; glycation inhibition assay	α-Amylase and α-glucosidase inhibition and antiglycation activity^142–144^.
*Morus alba* L.	STZ and high fat diet induced diabetic Wistar rat, STZ induced diabetic Wistar rat, STZ induced diabetic mice	Reduction in fasting blood glucose, Fasting serum insulin, homeostasis model of assessment-insulin resistance (HOMA-IR), glycated serum protein (GSP), and serum alanine transaminase (ALT) levels, improved serum insulin level^145–148^	α-Amylase inhibition assay	α-Amylase inhibition^149^.
*Murraya koenigii* (L.) Spreng.	STZ-induced diabetic Wistar rats; alloxan-induced diabetic male albino rabbits	Reduced fasting blood glucose, triglycerides, total cholesterol, LDL and VLDL levels, with increased HDL levels^150,151^.	α-Amylase and α-glucosidase inhibition assays	α-Amylase and α-glucosidase inhibition^152–154^.
*Musa balbisiana* Colla	STZ-induced diabetic Wistar albino rats; alloxan-induced diabetic Swiss albino mice	Reduced fasting blood glucose, serum total cholesterol, triglycerides, LDL and MDA levels; increased serum CAT, HDL and insulin levels^155–157^.	α-Amylase and α-glucosidase inhibition assays	α-Amylase and α-glucosidase inhibition^157,158,159^
*Nyctanthes arbor-tristis* L.	STZ and high fat diet induced diabetic Sprague–Dawley rats, normal ICR mice	Reduced fasting blood glucose level, nephroprotection, restored the normal architecture of the kidney and aorta tissue^160,161^	Enzyme assay	Alpha-amylase and alpha-glucosidase inhibition^160,161^
*Ocimum tenuiflorum* L.	STZ-induced diabetic Sprague–Dawley rats; STZ–nicotinamide-induced diabetic Wistar albino rats	Reduced fasting blood glucose levels and antihyperlipidaemic activity^162,163^.	α-Amylase inhibition assay	α-Amylase inhibition^164,165^.
*Oldenlandia corymbosa* L.	Alloxan-induced diabetic Sprague–Dawley rats	Reduced blood glucose, total cholesterol and LDL levels^166^.	α-Amylase and α-glucosidase inhibition assays	α-Amylase inhibition^167,168^and α-glucosidase inhibition^168^.
*Oroxylum indicum* (L.) Kurz	Alloxan-induced diabetic rats	Reduced blood glucose levels^169^.	α-Amylase and α-glucosidase inhibition assays	α-Amylase inhibition^170^ and α-glucosidase inhibition^170,171^
*Oxalis corniculata* L.	STZ-induced diabetic albino rats	Reduced blood glucose levels^172^.	α-Amylase and α-glucosidase inhibition assays	α-Amylase and α-glucosidase inhibition^173,174^.
*Phlogacanthus tubiflorus* Nees — No direct in vivo or in vitro antidiabetic evidence identified^*^				
*Phyllanthus emblica* L.	STZ-induced diabetic Wistar albino rats; STZ-induced diabetic mice; HFD-induced diabetic mice	Reduced blood glucose, HbA1c, triglyceride and total cholesterol levels; increased plasma insulin in STZ-induced diabetic animals and improved insulin resistance in HFD-fed mice^180–182^.	α-Amylase and α-glucosidase inhibition assays; glucose uptake assay	α-Amylase inhibition^183–185^; α-glucosidase inhibition^183,184^; increased glucose uptake^184^
*Physalis angulata* L.	Alloxan-induced diabetic rats; STZ-induced diabetic rats	Reduced blood glucose levels^186,187^and upregulated GLUT4 gene expression^187^.	Insulin-resistant C2C12 cell model; DPP-IV inhibition assay; α-amylase and α-glucosidase inhibition assays	Increased IRS-1 Tyr-612 and Akt Ser-473 phosphorylation^188^; DPP-IV inhibition^189^; α-amylase and α-glucosidase inhibition^190^.
*Piper longum* L.	STZ-induced diabetic Wistar albino rats	Reduced blood glucose, HbA1c, triglyceride and total plasma cholesterol levels; increased body weight, hepatic glycogen, plasma insulin and HDL cholesterol^191,192^.	α-Amylase and α-glucosidase inhibition assays	α-Amylase and α-glucosidase inhibition^193^.
*Psidium guajava* L.	Alloxan-induced diabetic albino rats; HFD–STZ-induced diabetic ICR mice	Reduced blood glucose, total cholesterol, triglycerides, LDL, glycated serum protein, creatinine and malondialdehyde levels; increased HDL levels^194–196^.	α-Amylase and α-glucosidase inhibition assays; glucose diffusion assay	α-Amylase and α-glucosidase inhibition^197,198^; inhibition of glucose diffusion^199^.
*Rubus moluccanus* L.				
*Scoparia dulcis* L.	STZ-induced diabetic Wistar albino rats	Reduced blood glucose levels; increased plasma insulin levels and activities of superoxide dismutase, catalase, glutathione peroxidase and glutathione-S-transferase^200,201^.	α-Amylase and α-glucosidase inhibition assays	α-Amylase and α-glucosidase inhibition^200,202^
*Solanum nigrum* L.	STZ induced diabetic Wistar rats	Reduced blood/plasma glucose, creatinine and BUN levels and kidney weight; improved Ca/Mg ratio and serum lipid profile, including total cholesterol, triglycerides, LDL, VLDL and HDL levels^203,204^	α-Amylase inhibition assay	α-Amylase inhibition^205^
*Solanum violaceum* Ortega	High fat diet induced Swiss Albino mice	Antihyperlipidemic, reduced triglyceride and total cholesterol^206^		
*Spondias pinnata* (L.f.) Kurz	STZ induced diabetic Wistar rats, Alloxan induced diabetic Sprague-Dawley rats, Normal Wistar rat	Reduced improvement in glucose tolerance, increase biosynthesis of insulin in the pancreas, antihyperlipidaemic, induce -cell regeneration^207–209^	Enzyme assay	Alpha-amylase and alpha-glucosidase inhibition^210^
*Syzygium aromaticum* (L.) Merr. & L.M.Perry	STZ induced diabetic Sprague-Dawley rats	Ameliorated postprandial hyperglycemia, down-regulated the increase of SGLT1 and GLUT2 expressions, increased hepatic and muscle glycogen concentrations, activity of glucokinase, hexokinase^211,212^	Enzyme assay	Alpha-amylase and alpha-glucosidase inhibition^212–214^
*Syzygium cumini* (L.) Skeels	STZ induced diabetic Wistar rats	Reduced glucose level, total cholesterol, triglycerides, and LDL-cholesterol, increased insulin level and HDL-cholesterol^215,216^	Enzyme assay	Alpha-amylase and alpha-glucosidase inhibition^217–219^
*Syzygium jambos* (L.) Alston	STZ induced diabetic rat	Lowered the blood glucose level^220^	Enzyme assay	Alpha-amylase and alpha-glucosidase inhibition^221,222^
*Terminalia arjuna* (Roxb. ex DC.) Wight & Arn.	Alloxan induced diabetic rats	Reduced blood glucose level, glucose-6-phosphatase, fructose-1,6-disphosphatase, aldolase; increased in the activity of phosphoglucoisomerase and hexokinase^223^	Enzyme assay	Alpha-amylase inhibition^224–227^
*Terminalia bellirica* (Gaertn.) Roxb.	Alloxan induced diabetic rats	Increased plasma insulin, C-peptide, and total serum protein, decreased total cholesterol, urea, triglycerides, uric acid, and creatinine^228,229^	Enzyme assay	Alpha-amylase and alpha-glucosidase inhibition^230^
*Terminalia chebula* Retz.	STZ induced diabetic rats	Glycosylated hemoglobin, beta cells number restored, improved glucose levels^231,232^	Enzyme assay	Alpha-glucosidase inhibition^233–235^
*Tinospora cordifolia* (Willd.) Hook.f. & Thomson	STZ induced diabetic rats	Plasma glucose, HBA1c, triglycerides, and total cholesterol decreased; increased hemoglobin, tissue glycogen, and HDL cholesterol^236^	Enzyme assay	Alpha-amylase and alpha-glucosidase inhibition^237,238^
*Trigonella foenum-graecum* L.	STZ-induced diabetic Sprague–Dawley rats; alloxan-induced diabetic Wistar rats	Reduced blood glucose, urea, creatinine, AST, ALT and triglyceride levels; increased protein levels; restored SOD and GST activities; reduced lipid peroxidation^239–241^	Enzyme assay	Alpha-amylase and alpha-glucosidase inhibition^242^
*Vitex negundo* L.	STZ induced diabetic Wistar Albino rats	Reduced blood glucose, plasma and tissues glycoproteins (hexose, hexosamine, fucose and sialic acid), increased insulin, catalase, reduced glutathione, and superoxide dismutase^243,244^	Enzyme assay	Alpha-amylase inhibition^244,245^

No direct antidiabetic studies were identified for Phlogacanthus tubiflorus; indirect evidence is available from studies on the congeneric species Phlogacanthus thyrsiformis^175–179^.

**Fig. 7:**
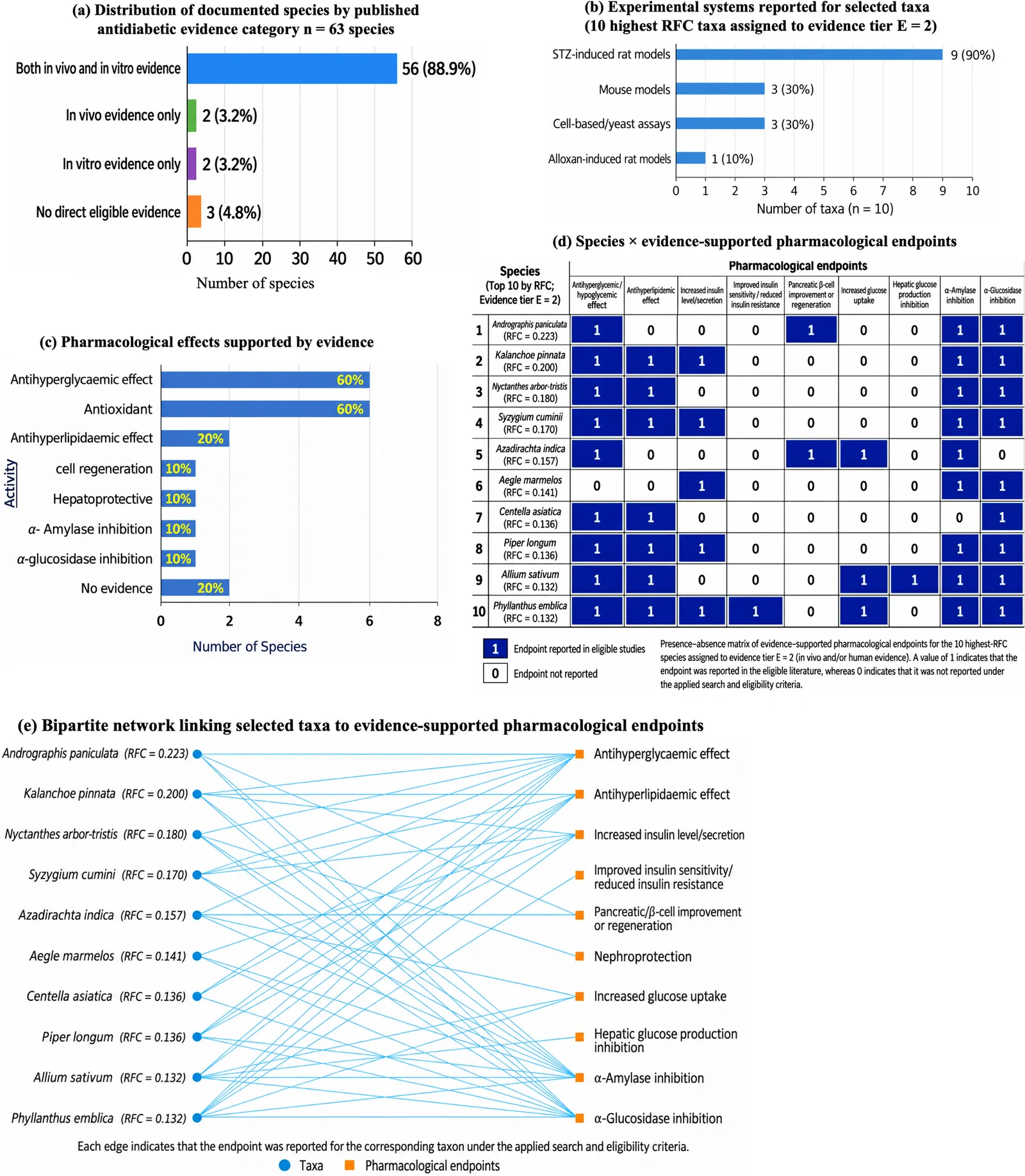
(**a**–**e**) Species-level pharmacological evidence profile of antidiabetic taxa documented in Biswanath district, Assam, India. (**a**) Distribution of all 63 documented taxa according to the eligible published antidiabetic evidence identified: both in vivo and in vitro evidence, in vivo evidence only, in vitro evidence only, or no direct eligible evidence. (**b**) Principal experimental systems represented among 10 selected taxa assigned to evidence tier E = 2. The taxa were selected by first restricting the dataset to E = 2 and then ranking them according to Relative Frequency of Citation (RFC) as an indicator of cultural salience. Bars indicate the number and percentage of taxa for which at least one eligible study used each experimental system. Categories are not mutually exclusive because an individual taxon may have been investigated using more than one system. (**c**) Frequency of evidence-supported pharmacological endpoints among the same 10 selected taxa; bars indicate the number and percentage of taxa for which each endpoint was reported. (**d**) Presence–absence matrix linking the selected taxa to their evidence-supported pharmacological endpoints, where 1 indicates that the endpoint was reported in the eligible literature and 0 indicates that it was not reported under the applied search and eligibility criteria. (**e**) Bipartite network representing the same taxon–endpoint associations; each edge indicates a reported association between a taxon and a pharmacological endpoint. Evidence was assessed at the species level and should not be interpreted as validation of the specific plant parts, preparations, doses or modes of administration documented locally.

Comparable regional and cross-cultural studies have shown that the status, perception, and use of medicinal plants vary across sociocultural settings, supporting interpretation of the present findings within the local biocultural context of Biswanath district^246^^247^,.

The accompanying literature synthesis (Table 3) and pharmacological evidence profile (Fig. 7) provide further insight into the current experimental landscape. Among the 10 highest-RFC taxa assigned to E = 2 and visualised in Fig. 7(b–e), STZ-induced rat models were most frequently represented, whereas alloxan-induced rat models, mouse models, and cell-based assays were less common (Fig. 7b)^248^^249^,. Within this selected panel, the most frequently reported pharmacological outcomes included antihyperglycaemic and antioxidant activities (Fig. 7c), whereas relatively few taxa were evaluated across a broader range of pharmacological endpoints or mechanistic pathways (Fig. 7d–e). This uneven distribution indicates that the available evidence for these selected taxa remains concentrated on a limited set of biological outcomes. From a translational perspective, the evidence base would benefit from more harmonised experimental designs, improved extract characterisation, inclusion of appropriate comparator drugs, and reporting of metabolically relevant secondary outcomes and safety parameters^250^^251^,. Such improvements would enhance cross-study comparability and strengthen the scientific foundation for future research on antidiabetic medicinal plants.

Taken together, the Fig. 6 prioritisation, Table 3 evidence synthesis, and Fig. 7 pharmacological profile provide a transparent basis for guiding subsequent research directions, including (i) strengthening standardisation and safety documentation for high-salience taxa with existing experimental support, (ii) advancing in vivo validation for taxa currently supported only by in vitro evidence, and (iii) investigating Innovation-zone taxa further through mechanistic and pharmacological studies.

### Limitations

This study has several limitations. Interview-based ethnobotanical data may be influenced by recall bias and courtesy or social-desirability bias; to minimise these effects, key information was cross-checked through repeat discussions and field observations where feasible. Men constituted 62.7% of the informants. Because the study was not designed for a balanced sex-stratified comparison and potential differences in age, education, experience, and recruitment pathways were not controlled, no conclusions can be drawn regarding gender-related differences in RFC, FL, or ethnomedicinal knowledge. Language- and translation-related effects were minimal because the field survey was conducted in Assamese by the first and second authors, both Assamese speakers, and the informants were able to communicate in Assamese; local residents assisted in clarifying community-specific terms where needed. As a cross-sectional study, it documents reported ethnomedicinal knowledge and practices during the survey period but does not assess changes over time or clinical outcomes. Pharmacological evidence was graded at the species level according to the highest eligible evidence identified and therefore may not correspond precisely to the plant part, preparation, dose, route of administration, or duration of use reported locally. Finally, RFC, UV, FL, and placement within the prioritisation zones reflect cultural salience, ethnomedicinal versatility, informant consensus, and the availability of published evidence; they do not establish therapeutic efficacy or safety and should not be interpreted as validation, recommendation, or endorsement of use.

## Conclusions

This study documented 63 medicinal plant species representing 57 genera and 39 families used for diabetes-related complaints in Biswanath district, Assam, based on ethnobotanical knowledge recorded from 440 informants. Diabetes-specific cultural salience analysis using RFC identified *Andrographis paniculata*, *Kalanchoe pinnata*, *Nyctanthes arbor-tristis*, *Syzygium cumini*, and *Azadirachta indica* as the most frequently cited taxa in local therapeutic practice. Overall UV across all recorded ailments distinguished widely used multipurpose household species, whereas diabetes-specific FL identified taxa showing stronger informant consensus for diabetes-related use.

By integrating ethnobotanical salience with literature-based evidence tiers derived from experimental studies, this study establishes a transparent and reproducible prioritisation framework linking community knowledge with available pharmacological support. The framework identified a high-salience Overlap group supported by experimental evidence, whereas none of the highly cited species fell within the Gap zone under the applied species-level search and eligibility criteria. Two species, *Hydrocotyle sibthorpioides* and *Alocasia odora*, were supported only by in vitro evidence, identifying priorities for future in vivo validation and more detailed pharmacological characterisation.

From practical and translational perspectives, the findings identify taxa requiring further investigation and underscore the need for harmonised experimental designs, improved extract characterisation and standardisation, rigorous dose–response evaluation, and systematic safety assessment. These steps are necessary before progression towards advanced preclinical or clinical evaluation. Particular caution is required for *Aristolochia assamica*: its documentation reflects reported local use and should not be interpreted as an endorsement, recommendation, or confirmation of safety, given the recognised nephrotoxic and carcinogenic risks associated with aristolochic acids in members of the genus *Aristolochia*. Sustainable harvesting also requires attention, particularly where roots, bark, or other destructive plant parts are used, to support long-term conservation and community-based resource management.

Overall, this study provides a documented account of antidiabetic medicinal plants together with an evidence-graded prioritisation framework. By connecting community knowledge with available pharmacological evidence, it provides a foundation for future phytochemical, pharmacological, toxicological, and translational research while supporting conservation-oriented and scientifically informed investigation of antidiabetic plant resources.

## Data Availability

All data generated and analysed during this study are included in this article. Additional raw data can be provided by the corresponding author upon reasonable request.
